# Cognitive status of patients judged fit for discharge from the post-anaesthesia care unit after general anaesthesia: a randomized comparison between desflurane and propofol

**DOI:** 10.1186/s12871-021-01287-9

**Published:** 2021-03-11

**Authors:** Cyrille Robert, Anne Soulier, Didier Sciard, Guillaume Dufour, Corinne Alberti, Priscilla Boizeau, Marc Beaussier

**Affiliations:** 1 Department of Anaesthesia and Critical Care, Clinique Mutualiste de Pessac, Pessac, France; 2 Department of Anaesthesia and Critical Care, St-Antoine Hospital. Assistance Publique-Hôpitaux de Paris, 75012 Paris, France; 3 Department of Anaesthesia, Institut Mutualiste Montsouris, 42 Boulevard Jourdan, 75014 Paris, France; 4 Unit of Clinical Epidemiology, Assistance Publique-Hôpitaux de Paris, CHU Robert Debré, University Paris Diderot, Sorbonne Paris-Cité, CIC-EC 1426 and, UMR-S 1123 ECEVE, 75019 Paris, France

**Keywords:** Cognitive, Anaesthesia, Desflurane, Propofol, PACU

## Abstract

**Background:**

The Aldrete’s score is used to determine when a patient can safely leave the Post-Anaesthesia Care Unit (PACU) and be transferred to the surgical ward. The Aldrete score is based on the evaluation of vital signs and consciousness. Cognitive functions according to the anaesthetic strategy at the time the patient is judged fit for discharge from the PACU (Aldrete’s score ≥ 9) have not been previously studied. The aim of this trial was to assess the cognitive status of inpatients emerging either from desflurane or propofol anaesthesia, at the time of PACU discharge (Aldrete score ≥ 9).

**Methods:**

Sixty adult patients scheduled for hip or knee arthroplasty under general anaesthesia were randomly allocated to receive either desflurane or propofol anaesthesia. Patients were evaluated the day before surgery using Digit Symbol Substitution Test (DSST), Stroop Color Test and Verbal Learning Test. After surgery, the Aldrete score was checked every 5 min until reaching a score ≥ 9. At this time, the same battery of cognitive tests was applied. Each test was evaluated separately. Cognitive status was reported using a combined Z score pooling together the results of all 3 cognitive tests.

**Results:**

Among the 3 tests, only DSST was significantly reduced at Aldrete Score ≥ 9 in the Desflurane group. Combined Z-scores at Aldrete Score ≥ 9 were (in medians [interquartils]): − 0.2 [− 1.2;+ 0.6] and − 0.4 [− 1.1;+ 0.4] for desflurane and propofol groups respectively (*P = 0.62*). Cognitive dysfunction at Aldrete score ≥ 9 was observed in 3 patients in the Propofol group and in 2 patients in the Desflurane group) (*P* = 0.93).

**Conclusion:**

No difference was observed in cognitive status at Aldrete score ≥ 9 between desflurane and propofol anaesthesia. Although approximately 10% of patients still had cognitive dysfunctions, an Aldrete score ≥ 9 was associated with satisfactory cognitive function recovery in the majority of the patients after lower limb arthroplasty surgery under general anaesthesia.

**Trial registration:**

Clinical Trials identifier NTC02036736.

## Background

Recovery from general anaesthesia is a complex process that can be broken down into several stages [1]. The “immediate wake-up” corresponds to the patient regaining consciousness and stable cardiovascular and respiratory conditions [2]. During this sequence, patients are extensively monitored in the Post Anaesthesia Care Unit (PACU) and supervised by specialized staff. Patients must reach a satisfactory level of recovery before being discharged. At present, the Aldrete score is the most commonly used score allowing patients to be discharged from the PACU and transferred to the hospitalization ward [3]. This score has a maximum of 10 points and it is considered that a score ≥ 9 allows patients to be discharged from the PACU under satisfactory safety conditions. The level of consciousness is one of the parameters of the Aldrete score. However, the Aldrete score is not tailored to address cognitive status recovery, which corresponds to the reappearance of fine psychomotor skills [4]. Cognitive functions encompass several different clinical features corresponding to distinct pathophysiological mechanisms [4–6].

Until now, cognitive dysfunctions have mainly been studied within a few days after surgery (usually 7 days) [4–6]. The pathogenesis of long-term cognitive dysfunction is multifactorial and relates mostly to neuronal inflammation and some aspects of cerebral vulnerability [4–7], that may even be independent of surgery and anaesthesia [8]. This is in contrast with immediate postoperative cognitive function, which is one of the components of the overall process of anaesthesia recovery, mainly related to the residual effect of anaesthetic agents [9].

Until now, the cognitive status of inpatients with an Aldrete score ≥ 9 when they leave the PACU to be transferred to the ward had never been reported. However, this parameter is of major importance because satisfactory cognitive recovery can allow patients to perceive and express eventual distress and to react appropriately to environmental stimulations when going back to their room. Furthermore, patients with residual memorization troubles are more prone to forget safety recommendations. Finally, cognitive status is clearly one of the components of patient’s satisfaction and global appreciation of the quality of recovery [10], as well as a relevant indicator of quality for the anaesthesia department [11].

It remains totally unknown how cognitive recovery follows the course of the reappearance of vital functions. Because the rate of emergence and immediate recovery differs between anaesthetic agents, and in particular between desflurane and propofol [12, 13], it can be hypothesized that cognitive recovery does not strictly follow the course of immediate recovery. The resumption of cognitive function at a given state of immediate recovery, according to the administered anaesthetic agents, has never been investigated.

The aim of this prospective randomized study was to compare the cognitive status of inpatients without pre-operative cognitive impairment, emerging either from desflurane or propofol anaesthesia at the time of PACU discharge (Aldrete score ≥ 9).

## Materials and methods

### Ethics and patients

This is a prospective single-center parallel randomized study conducted in St Antoine University Hospital (Assistance-Publique Hôpitaux de Paris). All methods were carried out in accordance with relevant guidelines and regulations and with CONSORT recommendations [14]. Ethical committee approval for this study (Ethical committee n° 13,887-P120702) was provided by the Ethical Committee: CPP (Comite de Protection des Personnes) Ile de France V, 184 rue du Fbg St Antoine, Paris, France (Chaiperson Prof JJ Boffa) on 2 April 2013. The study was registered in ClinicalTrials.gov (Clinical Trials identifier: NCT 02036736).

Patients less than 75 years old, undergoing hip or knee arthroplasty under general anaesthesia were eligible in the study. Patients with preoperative dementia (defined as a Mini Mental State evaluation (MMS) [15] of 24 or less), unable to perform the cognitive tests, or who received preoperative psychotropic agents, as well as obese patients (BMI > 35 kg.m^− 2^), patients with chronic alcoholism or addiction were not included.

Definitive eligibility was decided by the anaesthesiologist in charge of the patient on the pre-anaesthetic visit the day before surgery. The information was given and the consent form was signed at that time.

### Randomization

The randomization sequence was generated electronically with nQuery (version 6.01). Enrollment was done by clinicians at the operating room. After enrollment, treatment assignment was done with a secure study website (Cleanweb, Telemedicine Technologies, Boulogne- Billancourt, France) after verification of eligibility and consent status. The anaesthesiologist, responsible for enrollment and care at the operating room, was the only one knowing the allocation arm of the treatment. They were not involved in judgment criteria measurement thereafter. Access profiles to the e-CRF have been limited depending on the function of the investigator (evaluator vs anaesthesiologist).

Depending on the randomization, the anaesthesia maintenance was provided either by Desflurane (Group D) or Propofol in TIVA (Total Intravenous Anaesthesia) mode (Group P).

### Anaesthetic protocol

No anxiolytic premedication was given to the patient before surgery. Anaesthetic induction was performed with Propofol + Sufentanil + Atracurium. Patients had standard monitoring including depth of anaesthesia using the Bispectral (BIS®) index. Hypothermia was prevented by using warming blankets.

All patients were intubated and ventilated with a mixture of O_2_/N_2_O: 50/50%. Fluid loading was achieved with crystalloids and/or colloids depending on requirements.

According to randomization, patients were allocated to receive either Desflurane (Group D) or Propofol (Group P) for anaesthesia maintenance.

- Group D: Desflurane
  - Induction with a bolus of Propofol 2–3 mg/kg
  - Maintenance with a closed circuit of Desflurane with minimal alveolar concentration adapted to maintain a BIS value between 40 and 60.
- Group P: Propofol
  - Target controlled administration of Propofol at 2 and 4 μg/ml to be adjusted to maintain a BIS value between 40 and 60.

Supplemental boluses of Sufentanil and Atracurium were given as required. At the end of surgery (T0), the patient was transferred to the post-anaesthesia care unit (PACU). Tracheal extubation was carried out when the patient was conscious, with a respiratory rate above 12.min^− 1^, a core temperature > 36 °C, and without residual muscle weakness (residual curarization was assessed with Double-Burst Stimulation and antagonized if necessary).

Post-operative pain intensity at rest was evaluated using the Numerical Rating Scale (NRS) with 0 = no pain and 10 = maximal imaginable pain intensity. Post-operative analgesia was multimodal. The use of locoregional techniques for post-operative analgesia was encouraged (nerve block, trunk block +/− placement of a perineural catheter +/− wound infiltration). During the stay in PACU, if NRS ≥ 3, morphine was administered by titration (bolus of 1 mg IV repeated every 5 min until NRS at rest < 3).

After arrival in the PACU, the Aldrete score was checked every 5 min. Once the score of ≥9 had been attained, the cognitive tests were carried out for a second time.

The data from these tests was collected by the same investigator as the day before surgery in the case report form.

### Cognitive assessment

Preoperatively, the patient’s educational status was registered and a measurement of their anxiety level using the Amsterdam Preoperative Anxiety Information Scale [16] was determined.

Cognitive tests were performed by a blinded anaesthesiologist. The same anaesthesiologist made the preoperative and postoperative assessments. Cognitive tests were chosen on the basis of experimental validation and feasibility criteria. Because the process of cognition is multidimensional, it is mandatory to have several different tests exploring multiple distinct components [17, 18]. In this perspective, it was chosen to use, the Digit Symbol Substitution Test (DSST) [19], the Stroop color word interference test [20, 21], and the Visual Verbal Learning Test (VLT) [22]. The DSST was derived from the Wechsler adult intelligence scale: On a sheet of paper with a code indicating 9 letters corresponding to 9 digits, the patient must fill out horizontal rows with letters associated with empty cells in 90 s. In the word and colour interference test (Stroop color word interference test): the patient reads a list of words indicating colours (task 1), then gives the name of the colours in a list of colored rectangles (task 2). Finally, the patient must read words indicating one color with the word printed in a different colour (task 3). Patients have 45 s to complete each task. The number of correct words was counted. The VLT is a memory test that explores the immediate and long-term recall of a list of 10 words. All tests were affected in the same way by cognitive dysfunction.

In accordance with guidelines on how to conduct a multidimensional cognitive evaluation, an overall score that takes into account inter-individual variability and learning effect, in relation to the standard deviation of the population was calculated (Z score) [23].

$$ \mathrm{Z}\ \mathrm{score}=\frac{X_0-\overline{X_0}}{\sqrt{\frac{\sum \left({X}_0-\overline{X_0}\right)2}{n}}} $$

For any test, the average performance of a population is diminished by the pre-operative control value and divided by the standard deviation for the variation in the population, thereby giving a measurement of the magnitude of the deviation from the reference with appropriate sign. Signs were adjusted to assure that deterioration corresponds to a negative score for all tests.

The Z-scores for all tests can be summarized, calculating a combined Z-score that is calculated as the sum of all Z-scores divided by the standard deviation for the sum Z-scores. In our case, cognitive dysfunction was defined as a combined Z-score < − 2, or at least 2 Z-scores for single test parameters <− 2 [23].

### Criteria of evaluation

A primary criterion of evaluation was the difference on cognitive status between Desflurane and Propofol at Aldrete’s score ≥ 9. The main judgement criterion was Z combined scores at the time Aldrete score ≥ 9.

As secondary criteria, each test was analyzed separately in order to evaluate its sensitivity in screening for post-operative cognitive and psychomotor dysfunction. These analyses were performed using the Z test for each individual test but also by calculating the difference between pre- and postoperative assessments. Moreover, the number of patients with cognitive deterioration at Aldrete score ≥ 9 (regardless of the anaesthetic agent), intraoperative parameters, such as sufentanil consumption and BIS value was registered, as well as time interval between end of surgery and tracheal extubation, time interval between tracheal extubation and Aldrete score ≥ 9, pain intensity and opiate consumption in PACU. Finally, patient’s satisfaction was assessed with a 5 points categorical scale.

### Statistical analysis

Statistical analysis was performed according to published guidelines by the International Study of Postoperative Cognitive Dysfunction (ISPOCD) group [23].

Z scores between the two groups were compared by a t-test. In each group, Stroop, DSST and VLT scores between D0 and D1 were compared by paired t-tests or conditional logistic regressions when the assumption about symmetric distribution failed. The number of patients having cognitive deterioration at Aldrete score ≥ 9 according to anaesthetic agents was compared using Chi-2 test with Fisher exact correction.

Calculating the required sample size was complex as it depends on the tests selected and the way in which they are processed. To date, no published works have used the same battery of tests as in our evaluation context. Given the pilot data obtained in our department, the difference in combined Z-score was 1.1. Therefore a total number of 30 patients per group made it possible to highlight a difference of 20% (i.e. 0.9) in the Z score with an α risk of 5% and a β risk of 10%. Enrolled patients who did not participate further in the study were excluded for final analysis. Results are presented in medians [interquartils] or mean ± SD. The threshold for statistical significance was set at *P* < 0.05.

## Results

**Fig. 1:**
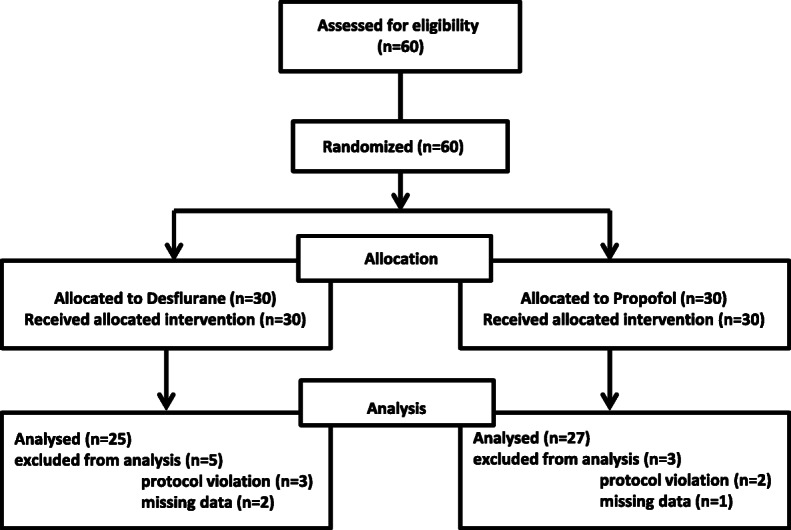
CONSORT Flow diagram

A total of 60 patients was enrolled and randomly allocated to Desflurane (*n* = 30) or Propofol (n = 30) subgroups. Five patients were excluded for final analysis in the Desflurane group (2 for missing data and 3 for protocol violation), and 3 in the Propofol group (1 for missing data and 2 for protocol violation) (Fig. 1).

**Fig. 2:**
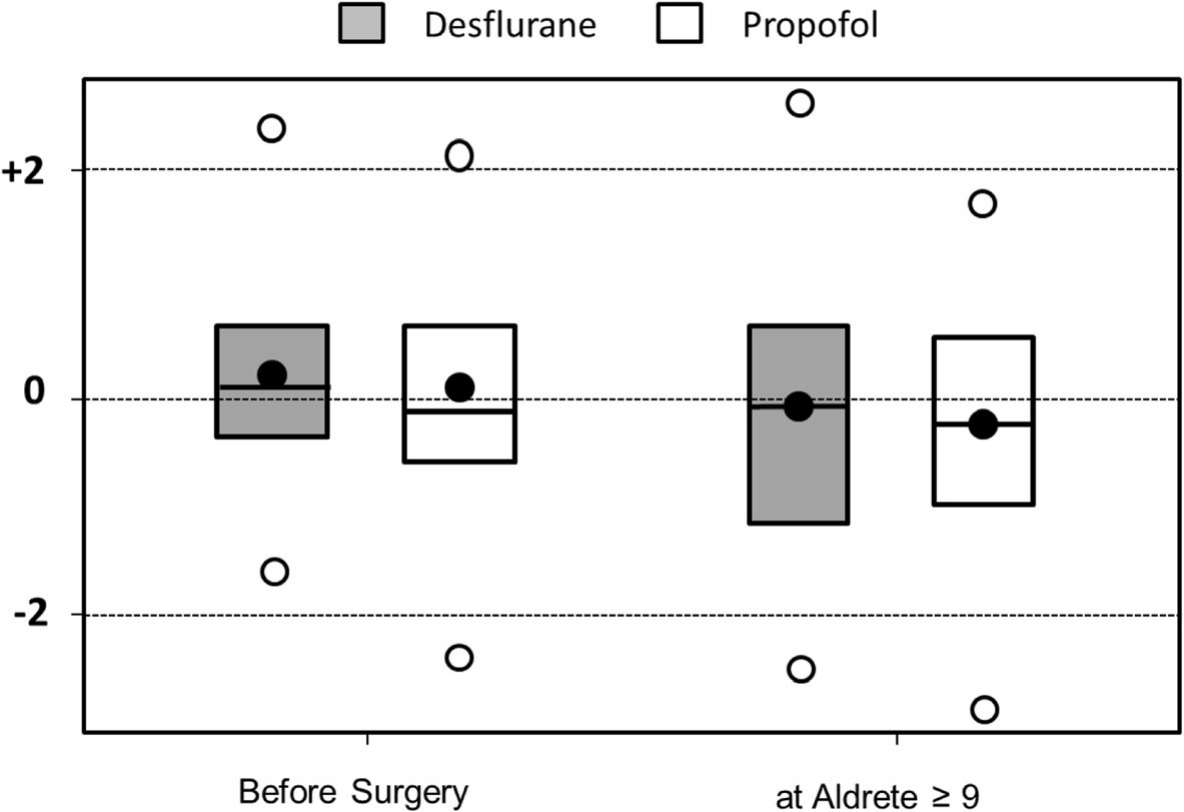
Evolution of combined Z scores between pre-operative and Aldrete score ≥ 9 assessments according to anaesthetic agent. Filled circles = means, horizontal lines = medians, box = interquartils, empty circles = extremes. No significant difference between groups

Demographic data are presented in Table 1 and did not statistically differ between groups. Intraoperative and postoperative data did not differ between the 2 groups (Table 2). Preoperative Mini Mental State (MMS) was 29 [27–29] and 29 [28–30] respectively in the Desflurane and Propofol groups. Preoperative anxiety, measured using the Amsterdam Preoperative Anxiety and Information Scale (APAIS) was 14 [7–17] and 15 [10–19] respectively in the Desflurane and Propofol groups (no significant difference). Preoperative cognitive functions did not significantly differ between groups (Fig. 2 and Table 3).

**Table 1:** Demographic data and information on procedures

	Desflurane ***n*** = 30	Propofol ***n*** = 30
Age (yrs)	68 [59–74]	70 [62–73]
Sex (M/F)	10/20	11/19
Height (cm)	165 [160–173]	164 [157–174]
Weight (kg)	77 [65–85]	70 [62–85]
BMI (kg.m^−2^)	28 [23–29]	27 [25–29]
Surgical procedures:		
Hip arthroplasty	16	16
Knee arthroplasty	14	14

Results in medians [interquartils]. No difference between groups

**Table 2:** Intraoperative and post-operative anaesthetic data

	Desflurane	Propofol	***P***
Duration of anaesthesia (min)	120 [98–280]	120 [84–320]	0.69
BIS at end of surgery	48 [43–54]	51 [41–58]	0.73
Time interval between end of anaesthesia and tracheal extubation (min)	10 [6–15]	10 [5–20]	0.45
Time interval between end of anaesthesia and Aldrete score ≥ 9	70 [52–90]	83 [65–110]	0.16
VAS at arrival in PACU (mm)	50 [0–80]	30 [0–70]	0.33
Morphine titration in PACU (Yes/No)	15/10	13/14	0.39
Morphine titration in PACU (mg) (among titrated patients)	10 [8–12]	10 [9–11]	0.78
PONV in PACU (n)	2	2	1

*BIS* Bispectral Index monitoring, *VAS* Verbal Analogic Scale, *PONV* Post-operative Nausea and Vomiting, *PACU* Post Anaesthesia Care Unit

No significant difference between groups

**Table 3:** Cognitive functions

	Desflurane			Propofol			***P*** *
	Pre-operative	Aldrete ≥ 9	***P***	Pre-operative	Aldrete ≥ 9	***P***	
DSST	29 [24/47]	25 [19/38]		22 [17/31]	25 [15/30]		
Mean difference		−8 [−9/− 1]	0.01		−1 [− 9/9]	0.45	
Z Score DSST		−0.4 [− 0.8/0.6]			−0.4 [−1.1/0]		0.46
Stroop test	117 [106/128]	120 [102/128]		115 [110/125]	112 [102/120]		
Mean difference		−1 [−7/0]	0.23		−3 [−11/2]	0.06	
Z score Stroop test		0.4 [−1.3/1.1]			−0.3 [−1.3/0.4]		0.37
VLT	6 [3/10]	6 [5/7]		6 [2/10]	6 [4/8]		
Mean difference		0 [−1/1]	0.81		0 [− 1/2]	0.76	
Z Score VLT		−0.1 [−0.6/0.4]			−0.1 [−1.1/0.9]		0.91

Results in medians [interquartils]

*DSST* Digit Symbol Substitution Test; *VLT* Verbal Learning Test

* statistical analysis comparing Desflurane and Propofol subgroups

Results of the 3 tests at Aldrete score ≥ 9 are presented in Table 3. Differences between preoperative tests and tests at Aldrete ≥9 are presented in Table 3. Only DSST in the Desflurane group was significantly reduced at Aldrete ≥9 in comparison to preoperative value. Combined Z-scores at Aldrete score ≥ 9 were − 0.2 [− 1.2;+ 0.6] (min = − 2.4; max = + 2.5) and − 0.4 [− 1.1;+ 0.4] (min = − 3.0; max = + 1.7) for the Desflurane and Propofol groups respectively (*P* = 0.62) (Fig. 2). The majority of patients did not present any cognitive dysfunction at Aldrete score ≥ 9. Only 3 patients in the Propofol group (combined Z-score = − 2.6, − 2.8 and − 3.0) and 2 patients in the Desflurane group (combined Z-score = − 2.1 and − 2.4) had a significant cognitive deterioration at the discharge time from PACU (*P* = 0.93).

Performing these cognitive tests was judged as “easy” or “very easy” for 18 patients in the Desflurane group and 21 patients in the Propofol group (*P* = 0.93).

## Discussion

In this study, it was found that the majority of patients had a satisfactory cognitive recovery at the time the Aldrete score achieved a value ≥9. Only 2 patients in the Desflurane group and 3 patients in the Propofol group had significant cognitive dysfunction when they were discharged from PACU. No difference was observed between desflurane and propofol anaesthesia, regardless of the time-interval to reach this score after the end of anaesthesia.

Cognitive recovery after general anaesthesia for lower limb arthroplasty surgery has been the subject of many studies [24, 25]. Regarding this topic, this surgical model is of particular interest because it uses highly reproducible procedures performed on elderly people. Recovery of cognitive function during the immediate postoperative period should be distinguished from cognitive deterioration (confusion or delirium) occurring days or weeks after the surgery and that are ascribed to other mechanisms than the residual effects of anaesthetic drugs [8]. In a previous study, delirium signs were observed in 31% of the patients 30 min after the end of the surgery, and were still present in 4% of them at PACU discharge [26]. In accordance, another report found a 15% incidence of delirium during the stay in PACU [27]. However, these articles focused more on behaviour (agitated or hypoactive signs rated on the Richmond Agitation and Sedation Scale) than on strictly cognitive status. The cognitive status at a predetermined level of awakening had never been investigated.

One of the major methodologic issues regarding cognitive assessment is to avoid confounding factors. Among other factors of influence, great attention was paid not to include patients with pre-operative cognitive deterioration [28]. Similarly, because the level of anxiety may interfere with cognitive evaluation, every patient had a pre-operative anxiety measurement.

Evaluation of cognitive function should be conducted according to several methodological recommendations [23]. It is recommended to use different tests exploring different components of cognitive skills. In the present study, it was decided to only use tests previously validated for the study of psychoactive drugs. Therefore we utilized the digit symbol substitution test (DSST) which is considered by psychometricians as a reference test for the evaluation of central coding disorders [17]. Like all coding tests, it explores particularly vulnerable functions in the postoperative period [29] and is able to discriminate recovery rates between different agents [30]. In the current study, DSST was the only test to be significantly impaired at Aldrete’s score ≥ 9. The Stroop color word interference (Stroop test) is an interference test between words and colours [20, 21]. This test is particularly robust for its reproducibility, independent of cultural factors, and explores specifically the functions of attention and concentration. The Verbal Learning Test explores the memory function which is very sensitive to the residual effects of halogenated agents as well as propofol [31, 32]. It should be noted that all these tests were considered easy to carry out by the majority of the patients.

Taking into consideration all of these methodological limitations, it was decided to strictly follow the usual recommendations on cognitive assessment [23]. In particular, the variability of the test measurements was analyzed in relation to the standard deviation of the population using the Z score. Z scores of all tests were thereby aggregated into a global “combined Z value”.

Cognitive evaluation is part of the global concept of post-operative quality of recovery [1]. As such, cognitive evaluation has usually been assessed at a constant time-interval after the end of anaesthesia, with 81% of patients judged as cognitively recovered at 90 min [33]. However, performing the cognitive evaluation at a constant time-interval from the end of anaesthesia could introduce some variability related to the different rate of elimination of anaesthetic agents and different conditions of immediate recovery. In this study, a different approach was chosen, allowing us to determine cognitive function at the same state of immediate recovery for every patient, regardless of the anaesthetic agents. It was chosen to search for the differences between desflurane and propofol. Desflurane is characterized by a rapid elimination rate [34]. Propofol is also characterised by fast elimination once administration has ceased. It is usually considered that desflurane allows for a faster recovery than propofol, even after short term exposure [35]. In the current study, no difference on cognitive status was found between desflurane and propofol at Aldrete score ≥ 9, while this was obtained sooner in the Desflurane group than in the Propofol group (not significantly different). This allows us to conclude that regardless of the anaesthetic agents, cognitive status gives the same level of performance at the same level of immediate recovery assessed by the Aldrete score. This result gives strong credit for the Aldrete score to be used as a means to determine a patient’s ability to leave the PACU under satisfactory safety conditions.

This study has some limitations. Cognitive testing very soon after general anaesthesia is somewhat problematic because of numerous confounding factors. In this study, great attention was paid to standardize anaesthesia and pain treatment. However, it cannot be excluded that other external or environmental factors, such as noise in PACU, might have interfered with our results. Regarding pain values, no statistical difference was observed between groups. Because of morphine titration, pain intensity on NRS was < 3 in every patient leaving the PACU. Similarly, morphine requirement did not differ between groups. It should be noted that morphine by itself has no influence on psychomotor performance in healthy subjects [36]. Potential bias induced by inter-individual variability and learning effects are common in cognitive evaluation. In this current approach, these factors were reduced by the use of Z score instead of direct average values. The single-center nature of this evaluation, as well as the small sample size, limits the ability to extrapolate the current results to other conditions. Finally, it should be noted that patients included in the final analysis were highly selected. In particular, patients with preoperative dementia or cognitive decline were excluded. In addition, because we wanted to focus on immediate cognitive recovery during awakening, we elected to limit the risk of variability induced by too wide ranges of ages or BMI. It is highly probable that results would have been different in another population.

In conclusion, no difference was observed in cognitive status at Aldrete score ≥ 9 between desflurane and propofol. Although approximately 10% of patients still have cognitive dysfunctions, an Aldrete score ≥ 9 was associated with satisfactory cognitive function recovery in the majority of the patients operated on lower limb arthroplasty surgery under general anaesthesia. This reinforces the clinical value of using Aldrete score to give the ability to be discharged from the PACU after general anaesthesia.

## Data Availability

Data collected for this study are available at Unit of Clinical Epidemiology, CHU Robert Debré, University Paris Diderot, Sorbonne Paris-Cité. Pr Corinne Alberti listed among the authors.
